# Motivations for consulting complementary and alternative medicine practitioners: A comparison of consumers from 1997–8 and 2005

**DOI:** 10.1186/1472-6882-8-16

**Published:** 2008-04-29

**Authors:** Fuschia M Sirois

**Affiliations:** 1 Department of Psychology, University of Windsor, 401 Sunset Ave., Windsor, Ontario, N9B 3P4, Canada

## Abstract

**Background:**

Use of complementary and alternative medicine (CAM), and especially CAM practitioners, has continued to rise in recent years. Although several motivators of CAM use have been identified, little is known about how and if the motivations for using CAM have changed over time. The purpose of the current study was to compare the reasons for consulting CAM practitioners in consumers in 1997–8 and eight years later in 2005.

**Methods:**

Surveys were displayed in CAM and conventional medicine offices and clinics in Ontario, Canada in 1997–8 and again in 2005, and self-selected participants returned the surveys by mail.

**Results:**

In 1997–8, 141 CAM consumers were identified from the 199 surveys returned, and 185 CAM consumers were identified from the 239 surveys returned in 2005. Five of the six CAM motivations were more likely to be endorsed by the 2005 CAM consumers compared to the 1997–8 CAM consumers (all *p*'s < .0001). In 1997–8 the two top reasons for using CAM were that CAM allowed them to take an active role in their health (51.8%), and because conventional medicine was ineffective for their health problem (41.8%). In 2005, the treatment of the whole person (78.3%) was the top reason for using CAM followed by taking an active role in one's health (76.5%). The 2005 consumers were less educated, had slightly more chronic health complaints, had been using CAM for longer, and were more likely to consult chiropractors, reflexologists, and therapeutic touch practitioners than the 1997–8 consumers. Otherwise, the socio-demographic and health profiles of the two groups of CAM consumers were similar, as was their use of CAM.

**Conclusion:**

Compared to consumers in 1997–8, consumers in 2005 were more likely to endorse five of the six motivations for consulting CAM practitioners. A shift towards motivations focusing more on the positive aspects of CAM and less on the negative aspects of conventional medicine was also noted for the 2005 consumers. Findings suggest that CAM motivations may shift over time as public knowledge of and experience with CAM also changes.

## Background

Interest in and use of complementary and alternative medicine (CAM) continues to rise in Canada and other developed nations, spurring interest into the motivations for CAM use. For example, in Canada, the use of CAM practitioners among the general population increased from 15% in 1994/5 to 20% in 2003 [1]. These rates are much lower than those generally found when CAM use includes both products and practitioners. One national survey found that in 2006, 54% of Canadians reported using a CAM product or practitioner in the previous year, an increase of 4% in the rate of use reported in 1997 [2]. As these studies indicate, use of CAM, and particularly CAM practitioners, is becoming more acceptable to the general Canadian population. The growing acceptance of CAM has also been noted in the U.S [3], Europe [4], and Australia [5].

To understand this trend, numerous investigations have sought to uncover the reasons why health-care consumers choose CAM. The motivations revealed by these studies are suggested to generally fall into two main categories: 1) reasons that highlight the perceived positive aspects of CAM, or "pull" factors and 2) reasons that focus on the perceived negative aspects of conventional medicine, or "push" factors [6]. A desire to take a more proactive role in one's health, and holistic health beliefs are among the more often cited "pull" motivations [7-9]. Dissatisfaction with aspects of conventional medicine, including unpleasant side effects [10], ineffective treatment [11,12], and aspects of the doctor-patient relationship [13], are common "push" motivations reported by CAM consumers.

Little is known, however, about how and if the motivations for using CAM have changed in recent years. With more people using CAM, the reasons that motivate the newest wave of CAM consumers may or may not be as salient as those that motivated CAM consumers in previous years. Once viewed as "unconventional" and limited in use to certain segments of the population, many CAM therapies are being viewed as more mainstream and acceptable treatment options by the general public. Knowledge regarding general shifts in the motives of CAM consumers can be useful for informing both practitioners and policy makers in their efforts to address current consumers' health care needs [14]. The purpose of the current study was therefore to compare the reasons for using CAM, and specifically CAM practitioners, in two Canadian general medical populations sampled from 1997–8 and eight years later in 2005, using the same CAM use and motivations questions. The two groups of CAM consumers were also compared to examine if they were similar in their demographic and health profiles, and in their general use of CAM.

## Methods

This study involved a secondary analysis of existing data. Ethics approval for this study was obtained from the University Research Ethics Board.

Two self-selected samples of medical-care seeking adults from the general population were obtained in 1997–8 and 2005. The recruiting methods used for both time points were identical, although the location of the recruitment sites differed. The 1997–8 sample was obtained from a large sized urban center in Eastern Ontario (estimated 1997–8 population of 750,000 based on 1996 census data for this public health unit area [15]), and the 2005 data from a mid-sized urban center in Southwestern Ontario (estimated population of 345,000 based on 2006 census data for this public health unit area [16]). For both sites, clinics were chosen from areas of low and high affluence, and included offices/clinics from the urban center as well as from the surrounding outskirts that were considered part of the public health unit census area. Accessibility to family physicians/general practitioners was slightly higher for the 1997–8 site, with an estimated 119 general practitioners (GP) per 100,000 people [15], versus 101 GP per 100,000 people for the 2005 site [16,17]. Although similar data for the availability of CAM practitioners for each site is not available, given the growth in the popularity of CAM between the two time points, it is reasonable to assume that there were a greater number of CAM clinics/offices per capita for the 2005 site.

**Figure 1:**
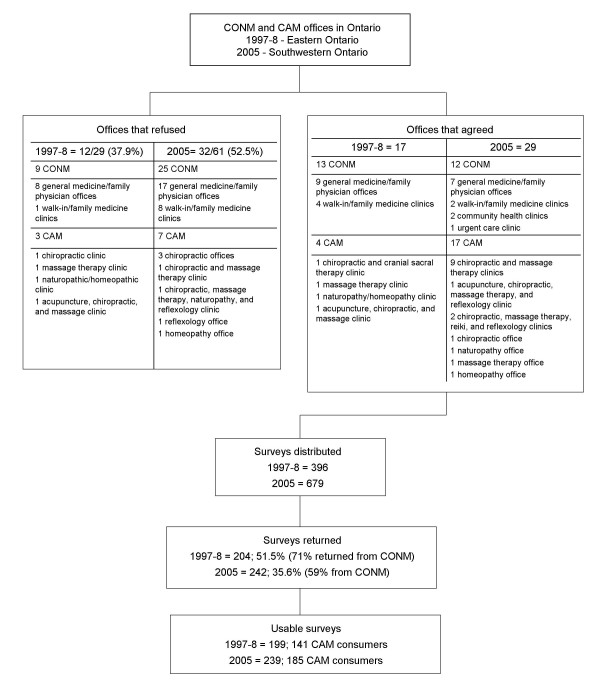
**Sampling frame for the 1997–8 and 2005 CAM consumers**. CONM = Conventional medicine; CAM = complementary and alternative medicine.

Questionnaires were made available to potential participants in several conventional medicine and CAM clinics or practitioner offices through a display box and sign advertising the study placed in the waiting room of each participating clinic or office. Figure 1 presents the number and types of clinics for each time point, including the specific types of conventional and CAM offices and clinics that participated in the study. Each office or clinic was staffed by one or more general medicine practitioners or CAM practitioners. Individuals interested in participating took the questionnaire from the display, completed it in the location of their choice, and returned it by mail in the postage paid return envelope.

The survey for each time point included questions about whether participants had ever consulted the CAM practitioners listed, with blank spaces to list any other CAM therapies or products they had ever tried. The CAM practices listed included chiropractic, massage therapy, homeopathy and naturopathy, acupuncture, reflexology, and reiki. Questions about how long they had been using CAM (under 6 months, under 1 year, 1 to 2 years, 3 to 5 years, or over 5 years), and how they use CAM (i.e., to supplement or replace conventional medicine) were also included. Questions about recent CAM use were also included. Participants indicated their agreement with six statements about their reasons for using CAM which began with the stem "I use complementary medicine/therapy because..." and were completed with different reasons. The statements were adapted from previous research identifying the main reasons for CAM use [18], and included motivations regarding positive aspects of CAM (2 items), dissatisfaction with aspects of conventional care (3 items), and a desire to find relief for a health problem (1 item). For the 1997–8 sample, the six reasons for CAM use were presented in a list and participants were instructed to indicate their agreement by circling any statements which reflected their own reasons for CAM use. For the 2005 sample the same six items were presented and participants indicated their agreement for each on a 6-point Likert-type scale with response options ranging from 1 (*strongly disagree*) to 6 (*strongly agree*). The response format was changed for the 2005 study so that a correlational analysis of the factors associated with the CAM motivations could be conducted for the larger study from which the data for this study was drawn.

Participants also completed questions about demographics and a checklist of acute and chronic health problems they had recently experienced at both time points.

### Statistical analyses

Participants for each time point were categorised as CAM consumers or non-consumers based on the responses to the CAM use questions. Individuals who indicated that they had ever tried any type of CAM, and 1) that they had consulted any CAM practitioner listed on the checklist in the past year, including specifying an "other" CAM practice, or 2) they had been using CAM for any length of time, were classified as CAM clients/consumers. Responses to the "other" category were screened to ensure that they qualified as CAM practices according to the four major domains of CAM practices suggested by the National Center for Complementary and Alternative Medicine (NCCAM) categories [19].

For each of the two samples, frequencies, counts, and means were calculated for the sociodemographic, health status, and CAM use variables to provide a descriptive profile of each sample. The CAM motivation Likert scale scores for the 2005 sample were recoded into disagree/agree scores so that they could be compared to the 1997–8 responses. A standard dichotomization of the Likert scores was conducted so that the disagreement scores (1 to 3; strongly disagree, disagree, mildly disagree) were converted to a score of 0 to indicate "no", and the agreement scores (4 to 6; mildly agree, agree, strongly agree) were converted to a score of 1 to indicate "yes". However, different response formats can have different effects on the survey response process and any associated response biases [20]. Therefore, it is possible that the increased number of response options on the 2005 Likert scaling of the items may have also introduced an acquiescence bias, that is a greater tendency and opportunity to agree with the items [21], compared to the dichotomous "yes/no" response options of the 1997–8 items. To compensate for this potential response bias a second more conservative recoding of the 6-point Likert responses was also conducted. In addition to the three "disagree" response options (1 to 3), the "mildly agree" response option was also recoded as "no", and only the "agree" and "strongly agree" options were recoded as "yes". Both the standard and the conservative recodings of the 2005 responses were compared to the 1997–8 responses on the CAM motivation items.

Differences between the 1997–8 and 2005 samples were examined with Fisher Exact tests for variables with 2 categories, and independent samples t-tests for continuous variables. Mann-Whitney U tests were used to examine differences between the two samples for ranked categories with a non-normal distribution, and Pearson Chi-square tests were used for non-ranked categorical data with more than 2 categories.

## Results

In 1997–8, 204 surveys were returned, and199 were considered usable. Based on the responses to the CAM use questions 141 CAM consumers were identified and included in the current study. In 2005, 242 surveys were returned, 239 were considered usable, and 185 participants were identified as CAM consumers and included in the current study. Among the "other" types of CAM listed, several participants listed "physiotherapist" as a type of CAM practitioner. Because the participants who gave these responses also indicated use of other valid CAM therapies, these responses but not the participants who gave these responses, were excluded from the analyses comparing the different types of CAM used.

As the participants were self-selected and obtained the questionnaires from the conventional medicine or CAM office waiting rooms, it is difficult to estimate exact response rates. Crude rates are estimated and presented in Figure 1 along with the rates of return based on office type (CAM or conventional medicine). For more details regarding the reasons for response rates see Sirois & Gick 2002 for the 1997–8 data collection [7], and Sirois & Purc-Stephenson 2008 for the 2005 data collection [11].

### Participants

The demographic and health status characteristics of each sample are presented in Table 1. Both the 1997–8 and 2005 were similar in most demographic and health characteristics, with significant differences noted for only two variables. The 1997–8 sample had attained a significantly higher level of education than the 2005 sample, and the 2005 sample had marginally more chronic health complaints.

**Table 1:** Demographic and health characteristics of the 1997–8 and 2005 CAM consumers.

	% (*N*) or *M *(*SD*)		
	
Characteristics	1997–8 *N *= 141	2005 *N *= 185	*p *value*
Sex (% female)^†^	77.9 (109)	83.2 (154)	0.26^(1)^
Age	42.3 (12.6)	41.4 (13.7)	
Range	19 – 80	15 – 86	0.54^(2)^
Caucasian ethnicity	93.5 (130)	94.1 (174)	1.00^(1)^
Employment status			
Full-time	55.3 (78)	53.5 (99)	
Part-time	19.1 (27)	16.2 (30)	
Unemployed/retired	15.6 (22)	23.2 (43)	
Disabled	9.9 (14)	7.0 (13)	0.31^(3)^
Education			
High school or less	8.5 (12)	23.8 (44)	
College/University	48.9 (69)	65.9 (122)	
Graduate school	42.6 (60)	10.3 (19)	<0.0001^(3)^
Relationship status			
Married/Living with spouse equivalent	65.2 (92)	63.6 (117)	
Separated/Divorced/Widowed	17.0 (22)	15.2 (28)	
Never married	17.7 (25)	19.6 (36)	0.98^(3)^
Number of acute health problems	3.62 (1.74)	3.34 (1.86)	0.60^(2)^
Range	0 – 8	0 – 9	
Number of chronic health problems	1.23 (1.07)	1.59 (1.24)	0.04^(2)^
Range	0 – 4	0 – 6	

* (1) Based on Fisher's Exact test, 2 sided, (2) based on an independent sample *t*-test, (3) based on a Pearson chi-square test, 2 sided.

† Analysis excludes one participant who self-identified as being transgender.

### CAM use

The 1997–8 and 2005 samples differed somewhat in their use of CAM (see Table 2). The 2005 sample had been using CAM for a slightly longer time than the 1997–8 sample, and had used marginally more different types of CAM than the 1997–8 sample. The majority of both samples indicated that they used CAM in addition to conventional medicine rather than using CAM alone and in place of conventional medicine.

**Table 2:** CAM use: 1997–8 and 2005 consumers

	% (*N*) or *M *(*SD*)		
	
	1997–8 *N *= 141	2005 *N *= 185	*p *value*
Length of time using CAM			0.03^(1)^
Less than 6 months	9.4 (13)	5.4 (10)	
6 months to 1 year	8.7 (12)	6.5 (12)	
1 to 2 years	16.7 (23)	11.4 (21)	
3 to 5 years	21.0 (29)	22.2 (41)	
More than 5 years	44.2 (61)	54.6 (101)	
CAM used to supplement *versus *to replace conventional medicine	89.8 (123)	88.1 (163)	0.72^(2)^
Number of different CAM ever tried	3.13 (1.68)	3.57 (2.18)	0.04^(3)^
Range	(1 – 7)	(1 – 13)	

* (1) Based on a Mann-Whitney U test, (2) based on Fisher's exact test, 2 sided, (3) based on an independent sample *t*-test.

**Figure 2:**
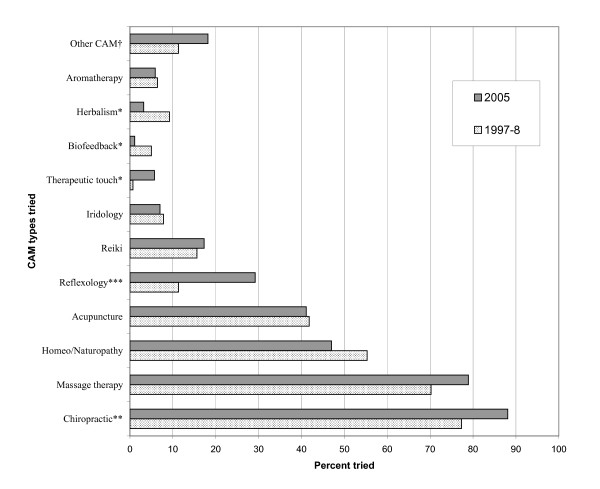
**The use of specific CAM practitioners and products by 1997–8 and 2005 consumers**. *p < 0.05, **p < 0.01, ***p < 0.001. † Other CAM practitioner types include cranial sacral therapists, Traditional Chinese Medicine practitioners, hypnotists, energy/pranic healers, osteopaths, spirit-based counselors, marma treatment, nutritionist, midwife/doula, accupressurist, shiatsu therapists, Alexander technique practitioner, colon irrigation, and music and art therapists. Other self-care CAM types included yoga, Tai Chi, Qi Gong, elimination diets, vitamin therapy, light therapy, ear coning, hydrotherapy, meditation, home remedies, and natural foods.

Figure 2 presents a comparison of the 1997–8 and 2005 consumers' use of specific CAM practitioners and products, and includes a listing of all the CAM practitioners that had been consulted, and any additional self-care CAM modalities. All participants who indicated the use of self-care CAM had also used at least one provider-based CAM. Significant differences were noted between the two samples in terms of the types of CAM practitioners and products that had ever been tried, although in both samples Chiropractic, massage therapy, homeopathy or naturopathy, and acupuncture were the four most commonly used CAM types (see Figure 2). Significantly more 2005 respondents reported consulting chiropractors, reflexologists, and therapeutic touch practitioners than the 1997–8 respondents.

### Reasons for using CAM

Table 3 presents the six reasons for using CAM and the rates of agreement for the 1997–8 and 2005 respondents, for each of the recoding methods (standard and conservative). Significantly more respondents from 2005 agreed with each of the six statements reflecting the reasons for using CAM when a standard recoding was used. However, even with a more conservative recoding which did not include "mildly agree" within the agreement category, there remained significant differences for five of the six CAM motivations examined, with ineffectiveness of conventional medicine as the only motivation that was endorsed by a similar proportion of CAM consumers for each time point. Difficulty communicating with one's physician was the least endorsed reason among both groups, although more respondents from 2005 endorsed this reason. The two top reasons for using CAM in the 1997–8 sample were that CAM allowed them to take an active role in one's health, and because conventional medicine was ineffective for their health problem. In 2005, taking an active role in one's health and the treatment of the whole person were the two top reasons for using CAM, with conventional medicine effectiveness as the third most endorsed reason.

**Table 3:** Reasons for using CAM in 1997–8 compared to 2005

	% (*N*) Agree		
	
I use complementary medicine/therapy because....	1997–8 *N *= 141	2005 *N *= 185	
		
		Standard recoding	Conservative recoding
1. Conventional medicine was not effective for my health problem.	41.8 (59)	67.2 (123)*	49.2 (90)
2. I believe that complementary/alternative medicine allows me to take a more active role in maintaining my health.	51.8 (73)	91.8 (168)*	76.5 (140)*
3. The conventional medicine treatment I received had unpleasant side effects.	9.2 (13)	55.7 (102)*	37.7 (69)*
4. I value the emphasis that complementary/alternative medicine places on treating the whole person.	36.9 (52)	92.4 (170)*	78.3 (144)*
5. I had difficulty communicating with my medical doctor (for example, he/she didn't understand my problem, didn't listen, etc.).	7.1 (10)	40.1 (73)*	22.0 (40)*
6. I am desperate to solve my health problem and I will try anything.	9.9 (14)	63.9 (115)*	45.1 (83)*

* Fisher Exact test of the difference between 1997–8 and 2005 score was significant at *p *< .0001.

## Discussion

A comparison of the motivations endorsed by CAM consumers in 2005 with consumers in 1997–8 revealed significant differences in the endorsement patterns. Consumers from 2005 were more likely to agree with five of the six motivations compared to the 1997–8 consumers. In addition, the top two reasons for CAM use in 2005 were focused primarily on the positive aspects of CAM (proactive role in health, holistic health perspective) rather than on the negative aspects of conventional medicine. In 1997–8 both a "pull" and a "push" motivation were among the top two reasons endorsed; in 2005 the top two reasons were both "pull" motivations.

The increase in the magnitude of the agreement with all but one of the listed CAM motivations in the 2005 CAM consumers relative to the 1997–8 consumers suggests that these are perhaps more salient motives for current CAM consumers than in the past. In particular, over three-quarters of the 2005 CAM consumers indicated that their CAM use was motivated by the positive aspects of CAM including the focus on treating the whole person, and the facilitation of a pro-active approach to health management. Increases in the dissemination of information about CAM (e.g., through the Public Health Agency of Canada) following the surge in popularity of CAM in Canada in the late 1990s, and the continuing trend towards health promotion and personal responsibility for health management, are plausible explanations for these findings. It is possible then that the shifting pattern of CAM motivations suggested by the current study may be a reflection of a greater knowledge and acceptance of CAM in 2005, as well as of shifts in societal values with respect to health and health care.

The results of the current study suggest that while both push and pull motivations are salient in the minds of past and present CAM consumers, motives related to the positive aspects of CAM may be gaining strength in the decisions made by current CAM consumers. Indeed, it is generally recognized that CAM decisions can be motivated by a variety of factors, and that different subgroups of CAM consumers may use CAM for different reasons [7,22]. Nonetheless, the debate regarding whether "push" or "pull" factors are more influential overall has continued, with CAM motive research in the 1990's suggesting that "push" factors such as dissatisfaction with the efficacy of conventional treatment were at least as important as "pull" factors in guiding consumers' CAM decisions [23-26]. Recent empirical evidence, however, favors the primacy of "pull" factors in CAM decisions [27-29], and is consistent with the trends suggested here. The posited shift in CAM motives over the past eight years towards a greater focus on the positive aspects of CAM may help practitioners and policy makers better understand the needs of today's CAM consumers, as well as improve understanding of what they expect from CAM practices. Rather than expecting treatments that are more efficacious than conventional care, this study suggests that current CAM consumers may expect and value aspects of treatment which provide empowerment and a more holistic view of health and healing that goes beyond simply managing symptoms.

Because the two groups of CAM consumers were remarkably similar in their socio-demographic and health profiles, the shifts in motivations and their degree of endorsement cannot be simply attributed to differences in sample characteristics or the different locations from which they were sampled. Age, gender, ethnicity, marital and employment status, and the number of acute health complaints reported were essentially the same for each group of consumers across the two time points. The one exception is that the 1997–8 consumers were more educated than the 2005 consumers. However, it is unlikely that this one difference can account for the significant increases in the level of endorsement of the five CAM motivations by the 2005 consumers. If anything, this distinction underscores the suggestion of other researchers that CAM use is no longer isolated to the highly educated as it once was [30].

There were also few differences between the two CAM consumer groups with respect to their experience with and way of using CAM. The majority of both groups of CAM consumers reported that they used CAM to supplement rather than replace conventional medicine, a finding that is consistent with other CAM research [31,32]. There was also a greater variety of CAM being used by the 2005 consumers, which may be a reflection of the growing popularity of CAM in recent years, and therefore the greater availability of information regarding different modalities and uses of CAM. Moreover, as certain types of CAM become more mainstream or acceptable, consumers may widen the scope of CAM that they would consider trying. The 2005 consumers also had slightly more years experience using CAM compared to the 1997–8 consumers, which may also account for their greater breadth of CAM use. This is not surprising given the surge of popularity of CAM in Canada in recent years, and especially during the late 1990's. Thus, the longer use of CAM reported by the 2005 sample could be a result of the time that had elapsed since 1997–8.

Differences in CAM use from 1997–8 to 2005 were noted for several different CAM practitioner types. More CAM consumers in 2005 reported consulting a chiropractor, reflexologist, or therapeutic touch than consumers in 1997–8. It is possible that this may be partly an artifact of differences in the number and type of CAM clinics sampled for each of the two time points, as a greater number of chiropractic and reflexology offices participated in 2005 compared to 1997–8. However, the increase in the use of chiropractic care noted is consistent with the results from national surveys which found an almost 50% increase in usage rates of chiropractic in Canada from 1998–9 to 2003 [1,33]. Interestingly, in 1999 provincial partial coverage of chiropractic care fees was reduced by over 30%, and then in 2004 chiropractic care was completely delisted from the Ontario's Health Insurance Plan (OHIP), leaving only those with Extended Health Care plans with some coverage for chiropractic fees [34]. Thus, the suggested increase in use of chiropractic care between the two time points occurred despite the extra fees that Ontario consumers had to pay for this CAM in 2005 relative to 1997–8 CAM consumers. One explanation is that chiropractic care has increased in its popularity and acceptability over the eight year time frame of the study, irrespective of any changes in provincial health care coverage.

With respect to reflexology, there is little comparative data available on historical changes in the use of this CAM modality that may similarly explain its greater use by the 2005 consumers, other than of course, differences in the clinic and site characteristics. That is, this difference could be explained by the increased availability of reflexology in the 2005 clinics/offices. Similarly, it is possible that the greater use of therapeutic touch (TT) in 2005 may be due to site specific characteristics rather than actual differences in utilization of this CAM modality over time. For example, TT is a popular and commonly recommended therapy at the 2005 site that is offered for free by several health organizations including the local Hospice and AIDS committee.

There are several methodological limitations in this study which warrant a cautious interpretation of the findings. First, the response options for the CAM reasons surveyed across the two time points were not identical, which may have had an impact on the endorsement pattern for the 2005 CAM consumers compared to the 1997–8 consumers. To address the issue of a possible acquiescence bias introduced by the Likert scale used in 2005, a conservative recoding was used in addition to the standard dichotomization of the 2005 responses, so that "mildly agree" responses were no longer considered as agreement with the CAM motivations. Even with this adjustment, there remained a higher level of endorsement from the 2005 sample for five of the six motivations, Furthermore, Nunnally [35] has suggested that including a balance of items that are positively and negatively worded would essentially eliminate response biases, including acquiescence. Thus, the fact that the six motivation items were evenly balanced between positively and negatively valenced items should have already contributed to reducing any tendency towards "yea-saying" or acquiescence before the application of a conservative recoding. Finally, there is some empirical evidence suggesting that two different formats of response options – a dichotomous scale and a six-point Likert scale – may be more or less equivalent when used to measure attitudes. For example, researchers [36] administered the same 20 item attitude scale with two different response formats – a dichotomous yes/no response, and a six point Likert scale ranging from strongly disagree to strongly agree – successively, with random assignment to one of the two counterbalanced presentation orders to test their equivalence. The degree of response similarity across the two response formats was high (approximately 80% shared variance), leading the researchers to conclude that the two formats were roughly equivalent and could be used interchangeably. Taken together, the conservative recoding, item balancing, and empirical evidence regarding the possible equivalence of dichotomous and 6-point Likert response formats suggests that the observed differences between the levels of endorsement of CAM motivations for each time point may indeed reflect actual shifts in the importance of these CAM motivations over time.

Because only a limited number of CAM motivations were examined, it is possible that other CAM motivations may not have changed or may have become less salient. Given the low agreement rates of the 1997–8 CAM consumers it is likely that there were other motivations for consulting CAM practitioners that were not assessed and which may have been more salient. Despite this, the motivations examined in this study had been identified by previous research as being among the top reasons for using CAM [18], and were clearly important motivations for the 2005 consumers. One explanation for the low endorsement rates in 1997–8 could be that these top reasons were previously identified in a sample of CAM consumers from the United Kingdom (U.K.) during the mid 1990's, a time when CAM knowledge and use was more widespread in the U.K. than what it was in Canada in 1997–8.

Another limitation involves the relatively small and self-selected samples of CAM consumers from each time point. However, the sampling methods employed for both time points were identical and the groups of CAM consumers obtained had very similar socio-demographic and health characteristics, providing further support for the proposition that the comparison of the motivations for CAM use between the two groups is valid. Although a larger number of CAM clinics were involved in the recruitment of the 2005 sample, the types of CAM practices offered at the clinics as a whole for each time point was roughly the same. Moreover, the increased number of CAM clinics in 2005 was due to the presence of a larger number of CAM offices at this site, relative to what was available in 1997–8. Given the smaller population of the 2005 site, it is likely that this difference reflects the increase in popularity and interest in CAM since 1997–8.

Finally, it is possible that the samples differed on some other characteristic that was not assessed, and that could explain the differences in CAM motivations found. Nonetheless, the pattern of CAM use found across the two samples mirrors that found in larger national surveys, suggesting that the two groups of CAM consumers and their motivations may be representative of the larger population of CAM consumers in Canada at each time point. For example, the four most used CAM practices at each time point in this study – Chiropractic, massage therapy, homeopathy/naturopathy, and acupuncture – were also found to be the most used CAM practices in the 2003 Canadian Community Health Survey which included responses from over 135,000 Canadians [1].

## Conclusion

In the current study, five of the six reasons for consulting CAM practitioners were more likely to be endorsed by the 2005 CAM consumers than by the 1997–8 consumers, suggesting that these motives may be more salient for current CAM consumers than in the past. The results also suggest that the motives for using CAM may have shifted over time to focus more on the positive aspects of CAM rather than rather than on the negative aspects of conventional medicine.

Increasing public awareness about the importance of attaining and maintaining wellness in Canada in recent years is one possible explanation for the suggested shift in motivations, as is the mainstreaming of particular CAM modalities. With little existing research on how CAM motives may shift as consumer awareness and use of CAM also shifts, this study offers a glimpse of how motivations for using CAM services may have changed since the late 1990's. Further research is needed to confirm the suggested trends in CAM motivations and whether they apply to CAM use outside of Canada.

## Competing interests

The author declares that they have no competing interests.

## Pre-publication history

The pre-publication history for this paper can be accessed here:


